# Connexins and Pannexins: Important Players in Tumorigenesis, Metastasis and Potential Therapeutics

**DOI:** 10.3390/ijms19061645

**Published:** 2018-06-01

**Authors:** Sheila V. Graham, Jean X. Jiang, Marc Mesnil

**Affiliations:** 1 Centre for Virus Research, Institute of Infection Immunity and Inflammation, College of Medical Veterinary and Life Sciences, Room 254 Jarrett Building, Garscube Estate, University of Glasgow, Glasgow G61 1QH, UK; Sheila.Graham@gla.ac.uk; 2 Department of Biochemistry and Structural Biology, University of Texas, Health Science Center, 7703 Floyd Curl Drive, San Antonio, TX 78229, USA; jiangj@uthscsa.edu; 3 STIM Laboratory, ERL 7003­CNRS/Building B36, University of Poitiers, 1 rue Georges Bonnet­TSA 51 106, 86073 Poitiers, CEDEX 09, France

**Keywords:** cancer, connexin, growth control, invasion, metastasis, pannexin, therapeutics

## Abstract

Since their characterization more than five decades ago, gap junctions and their structural proteins—the connexins—have been associated with cancer cell growth. During that period, the accumulation of data and molecular knowledge about this association revealed an apparent contradictory relationship between them and cancer. It appeared that if gap junctions or connexins can down regulate cancer cell growth they can be also implied in the migration, invasion and metastatic dissemination of cancer cells. Interestingly, in all these situations, connexins seem to be involved through various mechanisms in which they can act either as gap-junctional intercellular communication mediators, modulators of signalling pathways through their interactome, or as hemichannels, which mediate autocrine/paracrine communication. This complex involvement of connexins in cancer progression is even more complicated by the fact that their hemichannel function may overlap with other gap junction-related proteins, the pannexins. Despite this complexity, the possible involvements of connexins and pannexins in cancer progression and the elucidation of the mechanisms they control may lead to use them as new targets to control cancer progression. In this review, the involvements of connexins and pannexins in these different topics (cancer cell growth, invasion/metastasis process, possible cancer therapeutic targets) are discussed.

## 1. Introduction

**Figure 1:**
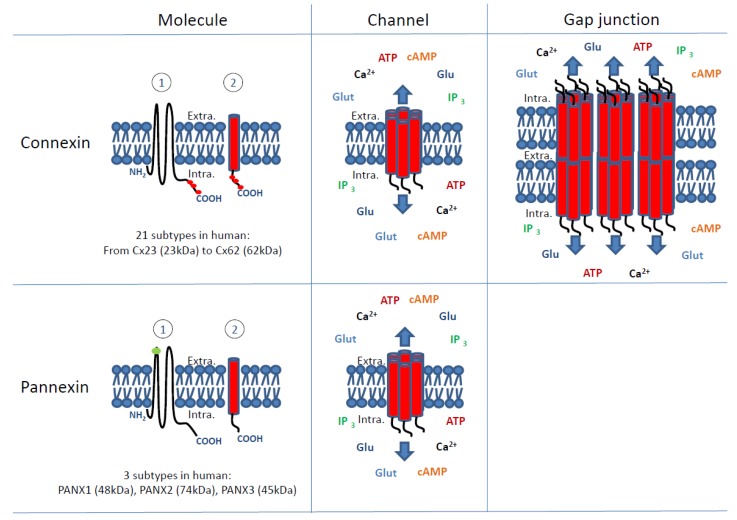
Connexin and pannexin molecules and channels formed by these molecules. As molecules, connexins (Cx) and pannexins (Panx) have similar topology with four transmembrane and intracellular (Intra.) NH_2_ and COOH domains. In the left panels, both kinds of molecules are shown in a “spread” way to distinguish their topology (1) and in a “condensed” way (2) to better represent as transmembrane subunits of channels (centre panels) and gap junctions (right panel). In humans, 21 subtypes of connexins have been characterized, which are differentially expressed in tissues [8]. They are named according to their expected molecular weight (kDa) from the smallest connexin (Cx23: 23 kDa) to the largest one (Cx62: 62 kDa). The best-known member of the connexin family is the connexin43 (Cx43) which is the most common in the organism. Only 3 pannexin subtypes are known in human (PANX1, PANX2, PANX3) [9,10]. Except for Cx26, connexins can be phosphorylated mostly at their intracellular COOH tail (red spots) [11]. The level of phosphorylation potentially modifies channel gating, interaction with intracellular or other membrane proteins (connexin interactome) and thus their function and life cycle [11,12]. So far, pannexins do not appear to be regulated by phosphorylation as connexins are but they are more characterized as potentially N-glycosylated (green spots) molecules at their extracellular (Extra.) domain. Both connexins and pannexins can aggregate to form hexameric transmembrane channels permitting the passive passage of ions (e.g., Ca^2+^) and small (<1–1.5 kDa) hydrophilic molecules such as nutrients (e.g., glucose: Glu), amino acids (e.g., glutamate: Glut), nucleotides (e.g., ATP) and second messengers (e.g., cAMP and IP_3_). Theoretically, connexin-made channels (connexons also called hemichannels) and pannexin-made channels (pannexons) are permeable to the same type of ions and molecules even if pannexons permeability has been mostly studied for ATP, Ca^2+^ and glutamate (Glut). Moreover, connexons from one cell can dock with connexons of juxtaposed cells forming intercellular channels aggregated in gap junctions which permit the direct intercellular transfer from cytosol to cytosol (gap-junctional intercellular communication, GJIC) of same ions and molecules as isolated connexons. So far, no pannexon-made gap junctions have been described in physiological/pathological conditions. The term connexon is mostly used to define the transmembrane unit of gap junctions. When isolated in the plasma membrane, connexons are usually called hemichannels and can open with various stimuli such as, for example, hypoxia. For clarity in the figure, putative phosphorylation sites (red spots) and N-glycosylated sites (green spots) are not shown in channels and gap junctions.

The majority of cancers in adults are solid tumours [1]. Whatever their tissue origin, those tumours are characterized by two fundamental properties, which are, first, an uncontrolled cell proliferation forming the tumour itself and then an acquired invasion capacity leading to the dissemination of cancer cells in the organism. Fifty years of investigation have shown involvement of gap junctions (GJs) or their molecular components, the connexins (Cxs), in these two fundamental characteristics of cancer progression [2,3,4]. More recently, it appeared that the involvement of Cxs could be complicated by the fact that they can act independently from the establishment of gap-junctional intercellular communication (GJIC). For instance, Cxs may be involved in these mechanisms through their interactome to modulate signalling pathways [5] or by acting as hemichannels (Hcs) mediating autocrine/paracrine communication [6]. This last activity may overlap with pannexins (Panxs) which are Cx-related proteins (Figure 1) [7].

Possible involvements of Cxs and Panxs in cancer progression and the elucidation of the mechanisms they control lead to their use as new possible targets to control cancer progression [13,14]. Here, we will review the involvement of Cxs and Panxs in these different topics, which are cancer cell proliferation, invasion/metastasis process and as possible targets for cancer control.

## 2. Connexins and Pannexins Involvement in Tumour Cell Growth

### 2.1. Connexins Involvement in Tumour Cell Growth

Shortly after their characterization, GJs were thought to be involved in growth regulation [15]. This assumption was the consequence of the possibility to estimate GJ functions through electrical coupling or diffusion of small hydrophilic fluorescent tracers [16,17]. By using such approaches, it rapidly appeared that cells derived from solid tumours (hepatoma, thyroid tumours, etc.) were not able to communicate through GJs [18,19]. These seminal studies introduced the notion that lack of GJ coupling could be a fundamental process in cancer leading to the formation of solid tumours by uncontrolled cell growth [15]. In other terms, growth regulation was the very first physiological role attributed to GJs and their mediation of a direct intercellular communication.

During the following decades, the involvement of GJIC in cancer cell growth regulation has been supported by a wide range of data. An early observation was about tumour promoter agents acting as inhibitors of GJIC [20,21]. This was observed in several models and reinforced the parallel between decreased GJIC and increased cell growth [22,23]. This parallel was extended to all kinds of phenomena able to inhibit GJIC such as cancer-causing viruses [24]. And such a phenomenon was so widely observed that it has been proposed that any GJIC inhibitor could be a potential tumour promoter [25]. If the tumour promoting effects of these chemicals were mostly known from in vitro studies, in some cases, GJIC inhibition effect could also be observed in vivo with transgenic mice exhibiting higher tumour susceptibility when defective for specific Cxs [26,27]. One of these best examples is liver since Cx32 gene knockout (KO) mice were shown to be more susceptible than wild-type mice to liver carcinogenesis after chemical treatments or even spontaneously [26]. This example was relevant to rodent and human situations for which liver tumours were correlated with lack of GJIC either by loss of expression or aberrant cytoplasmic localization of Cx32, respectively [28,29].

Conversely, strategies permitting the recovery of GJIC, by increasing Cx expression from non-communicating cancer cells, were expected to decrease cell growth. And indeed, globally this was the case as shown by approaches using chemical treatments or cDNA transfection. Chemicals known to be putative chemopreventing agents (flavonoids, carotenoids, retinoic acids, etc.) appeared to act on transformed cell lines by inducing GJIC and decreasing cell growth [30,31]. Cx cDNA transfection in GJIC-defective cancer cell lines brought similar conclusions that Cx expression is accompanied by decreased cell growth. This was observed in a variety of cancer cells (hepatoma, glioma, breast, etc.) in vitro and in vivo [32]. However, the type of transfected Cx was important since such an effect was mostly observed when the Cx of the normal tissue (before transformation or cancer progression) was re-expressed [33,34]. These results suggested that a recovery of GJIC is not sufficient by itself to have tumour suppressive effects but should be specifically controlled by the Cx subtype depending probably on the permeability capacity of the GJ it forms.

Thus, significant data accumulated over 50 years supported a similar conclusion. Whatever the models or the approaches (in situ detection of Cxs in tumours, cancer cell lines, chemical treatments, transgenic mice, cDNA transfection, etc.), the global conclusion is that Cx expression/GJIC is inversely correlated to cell growth. All these data have been analysed and synthesized in many reviews during past decades [2,3,4,32]. By considering all these observations, two kinds of molecular mechanisms can explain the involvement of Cxs in tumour cell growth regulation. The first one is to describe how Cxs, when present, can control cell growth. The second kind of molecular mechanisms, which are also needed for explaining the link between Cxs and cell growth, has to elucidate the origin of the lack of Cx expression or function which is observed in tumour cells. These are the two kinds of mechanisms that will be reviewed below.

#### 2.1.1. How Can the Presence of Connexins Regulate Cell Growth?

**Figure 2:**
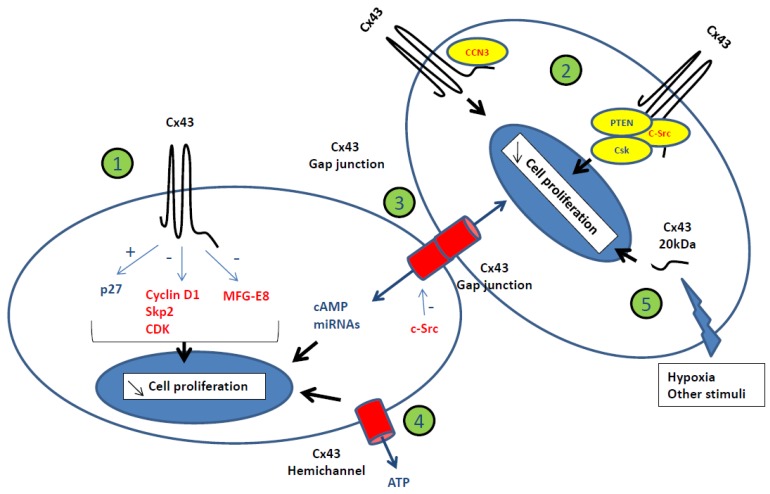
Connexin-mediated negative control of cell proliferation. Cx43 negatively regulates cell growth by acting differently on activators (red) and inhibitors (blue) of cell proliferation. This regulation is mediated through various mechanisms in which Cx43 acts by itself (1), as a sequestrator (2) of growth regulators (e.g., CCN3, PTEN, Csk, c-Src), as a mediator of GJIC (3), through hemichannel activity (4) or its 20 kDa carboxyl tail (CT)-domain (5). These various mechanisms act on the nucleus (thick black arrows) to decrease cell proliferation. Some of these mechanisms are mediated by hemichannel or gap-junction permeability (thick blue arrows). Positive (+)/negative (−) effects of Cx43 on cell cycle regulators (p27, Cyclin D1, etc.) and c-Src effect on Cx43 are also shown (thin blue arrows).

Most data attempting to elucidate how Cxs control cell growth came from Cx cDNA transfection in cancer cell lines. And from such approaches, whatever the cell types which were used (osteosarcoma, liver or lung carcinoma cells, etc.), a constant observation was that the increased expression of the original Cxs was followed by a longer G1 cell cycle phase slowing down the cell proliferation rate (Figure 2). A global analysis of these results suggests that this effect was the consequence of p27 accumulation [35,36]. From this common fact, diverse observations were made such as inhibition of enzymatic activity of Cyclin-dependent kinases (CDK) [36] and decreased amount of Cyclin D1 [37] and S-phase kinase-associated protein 2 (Skp2) [36,38,39]. To our knowledge, so far, no direct molecular link between Cx presence and the regulation of cell cycle has been demonstrated. Besides such an effect of Cxs on nuclear regulation of the cell cycle, it has been shown that Cxs can also act on the level of expression of growth factors. For instance, Cx43 re-expression but not Cx32, in C6 glioma cells is related to a decreased amount of milk fat globule-EGF factor 8 (MFG-E8) mRNA through an unknown mechanism (Figure 2) [40]. Therefore, Cx expression is mostly related to change of expression of growth factors or/and cell cycle regulators (p27, Cyclin D1, etc.). The most obvious scenarios for explaining how Cxs, when localized at the plasma membrane, can control gene expression might be through two major pathways. A first one would be through the Cx interactome by controlling growth transduction signalling and the second would be by diffusing growth regulators through GJs. Interestingly, as reported in the literature, both mechanisms have been observed and can explain the specificity of cell growth control induced by Cxs.

#### Gap-Junctional Intercellular Communication and Cell Growth Control

As mentioned above, the effect of Cx43 and Cx32 on cell growth has been extensively studied through various experimental models (cDNA transfection, transgenic mice, etc.) and appears to be specific. This specificity can be explained by their differential permeability which is illustrated by adenosine whose permeability is shifted from Cx32 to Cx43 channels by adding phosphate residues [41]. From such an observation, Cx channels appear as putative filters of intercellular signals that can be the consequence of the channel itself (diameter, amino-acid composition) or the configuration of the carboxyl tail (CT) which is sensitive to phosphorylation such as Cx43 channels closed by Src activation [42]. To our knowledge, a direct link between GJIC and growth regulation can be found in three situations. The first one is about the osteoblastic model in which extracellular growth stimulation induces the synthesis of second messengers that transit through GJs to activate extracellular signal-regulated kinase (ERK) and phosphatidylinositol-4,5-bisphosphate 3-kinase (PI3)/Akt serine/threonine kinase 1 (Akt) pathways. The translocation of ERK into the nucleus activates transcription factors that recognize a Cx-response element (CxRE) and induce osteocalcin and collagen I-1 expression [43]. Another example finally could explain the specific tumour suppressor effect of Cx26 on HeLa cells that was described two decades ago [33]. This effect seems to be the consequence of the maintenance of Cx26-mediated GJIC during the G2/M phase which permits intercellular cyclic 3′,5′-adenosine monophosphate (cAMP) redistribution able to delay the cell cycle progression (Figure 2) [44]. And more recently, it was shown that not only metabolites like cAMP could act as growth regulators passing through GJIC but also microRNAs (miRNAs) (Figure 2). As an example, the transmission of anti-proliferative effects from miR-124-3p-transfected to non-transfected glioma cells was mediated by GJIC [45]. Similarly, GJIC was shown to inhibit cancer cell growth by transferring miRNAs from endothelial cells in vitro [46]. And interestingly, it was observed that miRNA transfer can occur also by delivering from exosomes in which Cx43 facilitates the release of content into target cells [47].

#### Cell Growth Control Independent from Gap-Junctional Intercellular Communication

The specific effect of Cxs in cell growth control can come also from their cytoplasmic domains (internal loop and CT domain) which are unique in length and amino-acid sequences [8]. It has been known for a long time that these parts and in particular the CT domain, can interact directly with cytosolic/membrane proteins. Such interactomes have been mostly described for Cx43 for which about 40 different proteins have been identified as interacting ones [12,48]. From such observations, it became clear that the interactome may participate both to cell growth regulation by controlling channel permeability (i.e., channel closure due to Src-induced tyrosine phosphorylation of the Cx43 CT domain) or by modulating signalling pathways from the plasma membrane to the nucleus. For this last case, it has been postulated that the CT domain of Cxs could control, through sequestration, the translocation of putative transcription factors from the cytosol to the nucleus (Figure 2). Such a behaviour has been described for Cx32 with Discs large homolog 1 (hDlg1) in hepatocytes [49] and for Cx43 with CCN3 in rat C6 glioma cells [50,51]. In this last case, down regulation of Cx43 permits the translocation of CCN3 to the nucleus which activates cell growth (Figure 2) [50]. Such a situation can explain why glioma cell growth is higher when Cx43 expression is repressed and vice-versa. A similar situation has been shown for the transcription factor ZO-1–associated nucleic acid–binding protein (ZONAB) [52]. More recently, the tumour suppressive effect of Cx43 expression could be explained by the region 266–283 in the CT domain of Cx43 which is able to recruit PTEN and C-Terminal src kinase (Csk) to inhibit the oncogenic activity of c-Src (Figure 2) [53]. It is also possible that such a phenomenon could still happen when Cx43 is localized in the cytoplasm. Even if it has not been described yet, it would explain the down regulation of growth which was observed in human glioblastoma cells after transfection of Cx43 which was mainly localized in the cytoplasm [54].

In this last example, Cx43 signal was also detected in the nucleus of the cells [54]. The anti-proliferative effect associated with a nuclear signal of Cx43 is more intriguing. This effect could be due to the Cx43 CT domain since the transfection of that part only was followed by decreased growth in several cell types (HeLa, Neuro2a and HEK293 cells) [55,56,57]. It has been suggested that the Cx43 CT domain would then act as a transcription factor but this hypothesis has not been proven yet (Figure 2). However, a 20 kDa isoform which corresponds to the Cx43 CT domain is known to be translated in some cell types under certain conditions activated in cancer cells and hypoxia [58]. Its function is not known yet even if it has been shown to act as a chaperone protein for trafficking of Cx43 to the cell membrane [59] and for microtubule dependent mitochondrial transport [60].

Finally, to be complete, Cxs are known to form Hcs in the plasma membrane (Figure 1) [61]. Study of those Hcs has been growing this last decade, especially for Cx43 but their link with cell proliferation is still not obvious even if adenosine triphosphate (ATP) release and modulation of Ca^2+^ concentrations were correlated with decreased cell proliferation in several cell types [62]. In osteocytes, they have been found to be involved in suppression of breast cancer cell growth and bone metastasis using transgenic mouse models expressing dominant-negative mutants inhibiting either GJIC and/or Hcs [63]. With recent development of new research tools, such as Cx-interacting peptides, antibodies and dominant-negative mutants, the distinctive mechanisms of GJs versus Hcs, although still limited start to be elucidated. However, the action of Cx Hcs can still be confounded with Panx channels (Figure 1).

#### 2.1.2. What Does Prevent Connexin Expression or Function during Tumour Progression?

The expression of Cxs is often decreased in tumours whatever their origin [32]. Such a decreased expression may then participate to increase tumour growth by preventing the molecular mechanisms controlled by the presence of Cxs that were reviewed in the previous section. The molecular events leading to the disappearance of Cxs are not known precisely but could come from two mechanisms acting either at the transcriptional or at the post-transcriptional levels of Cx expression.

At the transcriptional level, similar to other genes which are shut down during tumour progression, Cx genes could be the target of epigenetic control. However, data about such a transcriptional control of Cx expression are not abundant in the cancer context even if it was suggested two decades ago [64]. In HeLa cells, silencing of the Cx43 gene was thought to be controlled by DNA methylation [65]. Loss of Cx32 function through hypermethylation is necessary for the development of renal cell carcinoma at the early carcinogenic process [66,67]. The CpG island hypermethylation level was associated with heavy smoking, poorly-differentiated tumour and low expression of Cx43 in non-small cell lung cancer [68]. More recently, hypermethylation of the Cx45 gene has been linked to its reduced expression in colon cancer [69]. This field of research is probably under investigated and would reveal if pursued that epigenetic phenomena are more involved than expected in the control of Cx expression.

At the post transcriptional level, Cx function can be regulated by ubiquitination, glycosylation, S-nitrosylation and in particular, phosphorylation of the CT domain. This has been mostly studied for Cx43 whose phosphorylation regulates GJIC through different mechanisms such as Cx trafficking, connexon assembly, channel gating and GJ degradation [11]. And indeed, in the cancer context, many oncogenes encode for kinases (i.e., c-Src) or proteins activating kinases (growth factor receptors) that are known to phosphorylate Cx43 and modulate its function [70]. As an example among others, epidermal growth factor (EGF) inhibits GJIC by inducing mitogen-activated protein kinase (MAPK)-mediated phosphorylation of Cx43 [71,72]. A similar effect has been observed for platelet-derived growth factor (PDGF) which activates MAPK and protein kinase C (PKC) pathways [73]. Interestingly, such a phosphorylation of the Cx43 CT domain establishes a direct link between growth stimulation and GJIC inhibition, which appears to be either the consequence of channel gating or Cx degradation [74].

Still at the post transcriptional level, an emerging field is about repression of Cx expression by miRNAs. For instance, mi-R-221/222 complex and miR-125b have been shown to downregulate Cx43 expression in glioma [75,76] or miR-20a in prostate cancer [77]. This field is still emerging and no doubt that it will be more involved in Cx gene regulation in future years.

Finally, the lack of expression or function of Cxs could be also theoretically the consequence of mutations affecting either the coding region of the Cx genes or their promoters. However, contrary to classical tumour suppressors (p53, Rb, etc.), such mutations have been rarely reported in the cancer context [32]. The most convincing result revealed a mutation affecting the Cx43 CT domain in human colon adenocarcinomas, which resulted in a restricted expression in invasive parts of the tumours [78]. To our knowledge, such an observation has not been confirmed. The fact that Cx mutations are not involved in human cancer is intriguing when considering their involvement in several human hereditary diseases [79]. So far, none of these diseases are known to be associated with a particular cancer susceptibility except for Cx26 mutations in the case of keratitis ichthyosis deafness (KID) syndrome which are associated with squamous cell carcinomas in 15% of patients [79]. The apparent general lack of association with cancer is probably the consequence of a lack of follow up of such patients.

### 2.2. Pannexins Involvement in Tumour Cell Growth

Originally, Panxs (3 members in mammals: Panxs1, 2 and 3) were identified as GJ proteins exhibiting homology with the invertebrate GJ proteins, the innexins [9]. Present in chordates, contrary to Cxs and despite a similar topology, they are not able to form functional GJs but form single membrane channels releasing autocrine and paracrine signals similar to Cx Hcs [10].

Data about a possible relationship between Panx expression and cancer progression or cancer cell growth are not so developed as they are for Cxs. In general, it seems that Panxs exhibit a so-called tumour suppressive effect similar to what is observed with Cxs. Such an analogy started during the last decade with the analysis of the brain cancer gene expression database REMBRANDT which revealed that the expression level of PANX2 and also PANX1 is positively correlated to post diagnosis survival of glioma patients [80]. To some extent, these observations were confirmed by the tumour suppressive effect induced both by Panx1 and Panx2 overexpression in rat C6 glioma cells in vitro and in vivo conditions [81,82]. In those cells, Panx1 expression had a wide range of anti-tumour activity by reducing in vitro cell proliferation, cell motility, anchorage-independent growth and tumour growth in nude mice. Interestingly, these effects, which are globally similar (except cell motility) with those observed after Cx43 transfection, were accompanied by an increased GJIC [81].

Similar observations have been obtained from skin where PANX1 and PANX3 levels are reduced both in human keratinocyte-derived basal cell carcinomas and squamous cell carcinomas [83]. This is in line with studies showing that those Panxs reduce growth of rat epidermal keratinocytes when overexpressed [84]. Such a growth inhibition was also observed for Panx3 in chondrocytes and osteoprogenitor cells by inhibiting the WNT pathway and via calcium-mediated regulation of p21 [85,86]. Recently, Panx3 was shown to inhibit the odontoblast proliferation through AMP-activated protein kinase (AMPK)/p21 signalling pathway and promote cell differentiation by bone morphogenetic protein (BMP)/Smad signalling pathway [87].

However, the situation is not so clear and probably depends on the cell type by considering melanocytes in which Panx1 expression is low whereas increased expression is correlated with melanoma aggressiveness [88]. More data are necessary before understanding the real involvement of Panxs in cancer cell growth control.

## 3. Connexins and Pannexins: Involvement in Tumour Metastasis and Microenvironment

### 3.1. The Process of Metastasis

**Figure 3:**
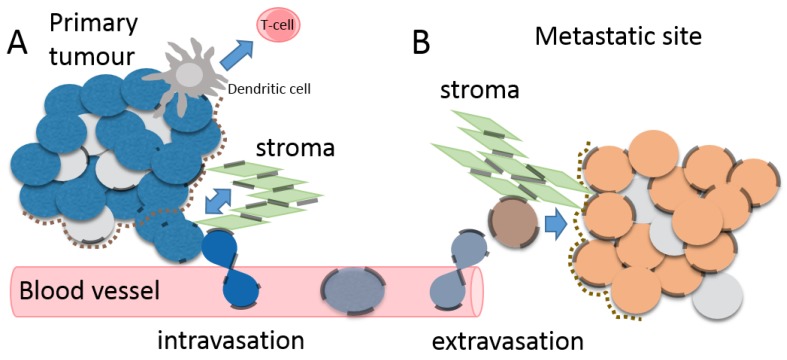
Gap-junctional intercellular communication in the tumour microenvironment and upon metastasis. (**A**) The tumour microenvironment consists of tumour cells (blue cells), non-tumour cells (light grey cells), immune cells including dendritic cells (dark grey cell) and CD8^+^ T-cells (pink cell), the basement membrane (brown dotted line) and the stroma (green cells). Tumour cells often display reduced gap junctions (transparent black lines) but can form heterotypic gap junctions with dendritic cells. Once they invade through to the stroma they can also form junctions with stromal cells. Upon intravasation into blood vessels tumour cells create gap junctions with endothelial cells lining the blood vessels; (**B**) Upon extravasation into a metastatic site, metastatic cells (orange cells) initiate gap-junctional intercellular communication with stromal cells and with other cells in the metastatic tumour microenvironment (light grey cells) and this may facilitate establishment of metastases. Depending on the site of metastasis, tumour cells may interact with cells of the immune system.

In order to become metastatic, a clone of cancer cells must acquire aggressive growth properties and/or stem cell-like properties and the tumour microenvironment can drive acquisition of migratory and invasive properties through epithelial to mesenchymal transition (EMT). In the majority of tumours, which are epithelial in origin, cells must be able to breach the basement membrane, invade into the stroma and into blood vessels (intravasation) that infiltrate the tumour site. In the vasculature, they will adhere to blood vessel walls and be transported to distant sites where they emerge from the circulation (extravasation) to initiated new tumours. Finally, establishment of metastatic tumours requires survival and growth in the new tissue microenvironment. During all of these processes metastatic cells must evade the anti-tumoral immune response (Figure 3).

### 3.2. Connexin Involvement in Tumour Metastasis and Microenvironment

#### 3.2.1. The Role of Connexins in Cancer Progression

Cxs can change expression levels, be re-localized [89,90,91,92] and/or exhibit altered phosphorylation upon progression to invasive tumour (Table 1) [93]. The resulting loss of functional GJs could alter tumour cell interaction with its microenvironment and promote EMT and migration from the primary tumour. Conversely, Cx expression can facilitate intravasation and adhesion to endothelial cells, enabling increased survival in the circulation. There is also evidence that Cx expression promotes exit from blood vessels into the metastatic site, where GJIC may be reinitiated [94] (Figure 3). However, Cxs may both promote tumour cell dormancy and cell survival, at metastatic sites. These effects may be reliant on tumour/stromal interactions and cooperation between invasive/metastatic cells and GJ formation in the tumour microenvironment and are likely to be Cx type, tumour type and cancer-stage-specific.

#### 3.2.2. Invasion and the Local Microenvironment

E-cadherin is required for invasion in EMT and its loss is a marker of tumour progression. During invasion, cells display decreased GJIC, modification of cell-matrix interactions and acquisition of proteolytic properties to degrade the basal laminal proteins. Following this, altered stromal cells and microenvironment facilitate the motility of invasive cells through the extracellular matrix. All of these processes could be potentially altered by changes in Cx expression. For example, transfection of poorly coupled mouse epidermal cells with an E-cadherin expression construct increased GJIC [117]. Conversely, in prostate cancer cells, Cx43 levels correlated with levels of the transcription factor Snail-1 that inhibits expression of E-cadherin to promote EMT [118]. High levels of Cx43 and Snail-1 resulted in increased tumour cell invasion and Cx43 was downregulated upon Snail-1 silencing and vice versa. In keeping with these findings of a Cx43-Snail-1 axis controlling tumour cell behaviour, Cx43 expression could reverse A549 lung tumour cell resistance to the chemotherapeutic drug cisplatin by downregulating E-cadherin and EMT, while siRNA depletion of Cx43 initiated EMT [119]. Melanoma, breast, prostate and gastric cancers all display upregulated Cx43 and Cx26 in invasive lesions and metastases (Table 1) [101,103,104,112,115].

#### 3.2.3. Promoting Metastasis: Connexins and Cell Motility

Early studies revealed that HeLa cervical cancer cells overexpressing Cx43 gained invasive properties in a chicken heart spheroid assay [120]. In a mouse melanoma model of metastasis following subcutaneous injection, clone F10 was less metastatic than the high Cx26-expressing clone BL6 but became as metastatic as BL6 upon Cx26 overexpression and BL6 cells expressing dominant negative Cx26 showed reduced metastatic potential [116]. γ-irradiation of C6 glioma cells induced Cx43 expression and increased ERK signalling and cell migration and a high Cx43 expressing clone displayed increased motility and invasion [121]. Conversely, knocking down Cx43 abrogated p38 MAPK activation and radiation-induced C6 cell migration [122]. Although GJIC was decreased upon Cx43 small interfering RNA (siRNA) depletion in the high Cx43 expressing C6 cells, GJ inhibitors did not alter motility indicating that Cx43 itself was responsible for the pro-metastatic effects [121]. Similarly, in a six-cell model of hepatocellular carcinoma, following injection into the tail vein of mice, only those lines with high metastatic potential formed foci in the lungs of the animals and this was reversible by depletion of Cx43 expression [113]. Another study found that blocking GJIC in GL15 glioblastoma cells increased motility in an in vitro 3D culture model [123]. However, blocking heterologous GJIC in ex vivo brain tissue by carbenoxolone reduced cell migration [123].

Cxs can facilitate adhesion of migrating cells to the endothelial layer of blood vessels and/or to specific distal sites (Figure 3). For instance, metastatic lung cancer cells could adhere to endothelial cells through GJs [124] as could metastasis-enabled melanoma cells ectopically expressing Cx26 in in vitro cultured vein segments [116]. In the case of colon cancer cells, conditioned medium from primary tumour cells enhanced phosphorylation of Cx43 and GJ formation between tumour and endothelial cells via the molecular chaperone heat shock protein 27 (HSP27), while metastatic colon cancer cells induced expression of Cx32 through action of the chemokine receptor CXCR2 [125]. Breast cancer cells that formed functional Cx43 GJs with endothelial cells facilitated migration out of the endothelial layer in in vitro culture [126] implicating Cx43 in the extravasation phase of metastasis. In zebrafish and chick embryo models, breast cancer and melanoma cell metastasis was dependent upon Cx43 and Cx26 to initiate brain metastatic lesions in association with the vasculature. Inhibition of Cx43-mediated GJIC inhibited extravasation, as did knock down of the EMT transcription factor twist [127].

#### 3.2.4. Involvement of Gap Junctions and Hemichannels in Metastasis

Apparently contradictory effects of Cx43 in metastasis have been observed in different studies. When a functional null mutant Cx43 mouse line (G60S: that also has dominant negative effects on endogenous Cx43 activity) was crossed with erythroblastic leukemia viral oncogene homologue (ErbB) overexpressing mice [128], there was delayed onset and fewer and smaller primary breast tumours than in wild type mice but increased metastases to the lung [128]. In contrast, Cx43 overexpression in highly metastatic lung cancer cells reversed the metastatic tumour phenotype [129] but decreased Cx43 gene expression yielded breast cancer cells with increased metastatic potential [130,131]. In a two-cell model of prostate cancer, overexpressed Cx43 was present only in the cytoplasm and repressed proliferation, adhesion and invasion of normally invasive PC-3 cells. In contrast, overexpression of Cx43 in poorly metastatic LNCaP cells, re-established GJIC and increased bone metastasis in mice [132]. Stable overexpression of Cx43 in the MDA-MB-435 breast cancer cell line did not alter GJIC, invasion or migration in vitro. However, when injected into mice, the cells exhibited a reduced growth rate and fewer lung metastases [106]. This phenomenon was found to be GJIC-independent and it was suggested that it could be related to reduced N-cadherin expression, which would inhibit EMT. In another study, GJIC was restored in the same metastatic breast cancer cell line upon ectopic expression of the breast cancer metastasis suppressor gene BRMS1 [130]. The BRMS1-expressing cells showed increased levels of Cx43 but reduced Cx32, leading to loss of GJIC between breast cancer cells and between them and breast epithelial cells [130]. An in vivo murine study revealed that metastatic breast cancer cells in the bone formed more active GJs with osteoblasts than with themselves and BRMS1 expression increased homotypic GJIC. The breast cancer cells with increased heterotypic, relative to homotypic, GJ channels with osteoblasts were more metastatic than those that did not [105]. This suggests that the relative percentage of homo- and heterotypic GJ channels in tumour cells can influence metastasis. Moreover, it suggests that heterotypic GJs could be an important survival mechanism of tumour cells in the metastatic tumour microenvironment. It can be concluded that the precise timing of elevated or reduced Cx expression could be key to any effects during tumour progression.

Compared to GJs changes in Hc activity can produce different effects in metastasizing tumour cells. In a bone metastatic clone of MDA-MB-231 breast cancer cells, decreased Cx26 and Cx43 levels correlated with metastatic potential partly through alterations in Hc activity [107]. Similarly, a recent study reported suppression of breast cancer cell metastasis to the bone through osteocytic Cx43 Hcs [63]. Drug or mechanically-induced opening of Cx43 Hcs to release ATP from osteocytes led to inhibition of invasion and migration of the cancer cells. Analysis of a dominant negative Cx43 mutant that blocks GJs but not Hcs, revealed that Cx43 Hcs protected against tumour progression and metastasis [63]. The precise role of Cxs in tumour progression and metastasis might depend on the nature of the tumour, the properties of the cancer cell itself, the site of metastasis and the possibility of forming functional GJs at that site. It is clear that the tumour microenvironment drives cancer metastasis and Cx43 seems to stimulate growth of brain metastases after extravasation and tumour vasculature remodelling [133]. Protocadherin 7, a brain-specific cadherin, promoted Cx43-GJ assembly between breast and lung tumour cells and astrocytes. These GJs allowed cyclic guanosine monophosphate (cGAMP) to activate the stimulator of interferon genes (STING) pathway in astrocytes to induce an interferon response. The resulting changes in cell signalling could enhance growth of metastatic cells [133].

#### 3.2.5. The Tumour Microenvironment

The tumour microenvironment, whether at the primary or secondary sites, is key to tumour cell survival and tumour progression [134]. In agreement with the hypothesis that Cxs control the microenvironment, Cx43-transfected glioma cells, which formed GJs with astrocytes in the striata of rats, were able to disseminate throughout the brain parenchyma. Cx43 itself, unlinked to GJIC, was shown to induce adhesive properties in the malignant glioma cells, which formed aggregates and were more invasive [135]. Also in rats, formation of GJIC with fibroblasts in co-culture stimulated prostate cancer cell migration [136,137]. However, Cx32 expression in metastatic renal cancer cells caused abrogation of invasive capacity via inactivation of c-Src signalling [138]. Tumour-associated immune cells are components of the tumour microenvironment. Heterotypic Cx43-GJs between tumour cells and dendritic cells can transmit melanoma antigenic peptides leading to activation of cytotoxic T-cells in vitro [139]. In vivo demonstration of Cx43-GJ transmission of antigenic peptides between antigen presenting cells has also been demonstrated [140]. GJ transmission of miRNAs between immune cells in the microenvironment and tumour cells is also expected to be a major regulator of metastasis because of the key role of many miRNAs in tumour suppression, while others can promote tumour progression [141].

### 3.3. Pannexins and Metastasis

The potential role of Panxs in metastasis is relatively unexplored. However, high levels of PANX1 mRNA were associated with metastatic spread in a two-cell model of hepatocellular carcinoma [142]. A key advance in understanding the role of Panxs in metastasis came from a study of the isogenic melanoma cell lines, F10 and BL6, mentioned previously. PANX1 levels were greatest in the most metastatic BL6 line [88]. PANX1 knock down reverted BL6 cells to a more normal melanocyte phenotype and these cells had reduced levels of vimentin and β-catenin, both markers of melanoma progression [88]. Importantly, in vivo data in a chick embryo xenograft model showed that reducing PANX1 expression reduced tumour growth and metastasis to the liver. A recent RNASeq analysis of breast cancer cells with different metastatic capacities revealed that cell lines with high metastatic potential had significantly enriched mutant mRNA encoding a N-terminal truncated PANX1 channel [143]. Truncated PANX-1, in association with wild type PANX1, seemed to confer a gain-of-function to channel activity and was found to promote metastatic cell survival. This appeared to be due to protection of tumour cells exiting the microvasculature via restrictive spaces between endothelial cells by enhancing ATP release from the Panx channels stimulated by mechanical deformation and abrogation of cell death [143]. In melanomas, P2X7/PANX1 channel activity has been linked to regulation of the NLRP3 inflammasome, which can result in release of pro-inflammatory, tumour promoting cytokines. Downstream effects on the tumour microenvironment could stimulate tumour growth and invasion. Of course, like Cxs, Panxs might also be found in future to repress tumour progression and metastasis.

## 4. Connexin and Pannexin Channels in Potential Cancer Therapeutics

### 4.1. Connexin Channels in Potential Cancer Therapeutics

The usefulness of Cxs and GJs as potential therapeutic targets for treating cancer has been studied for over four decades [4,144,145]. In recent years, several approaches have been developed in animal models to determine treatment modality by manipulating Cx channels. Although preclinical studies targeting connexin channels are still in their infancy, they hold great promise as de novo targets for cancer treatment.

#### 4.1.1. Chemical Compounds in Modulating Connexins and Potential Cancer Therapy

Major attempts have focused on enhancement of GJIC function due to its impairment in primary cancer cells. Multiple chemical compounds have been used (e.g., retinoids, vitamin D, carotenoids, cAMP and lovastatin), which can fully or partially reverse the deficiency of GJIC in tumorigenic cells [146]. Lypopene, a carotenoid stimulates GJIC and Cx43 expression and inhibits the growth of the breast cancer MCF-7 cell line [147]. Extracts from the zooxanthellate jellyfish that show antioxidant activity exhibit higher levels of GJIC and cytotoxicity in MCF-7 cells than human epidermal keratinocytes [148].

An experimental approach was developed that killed tumorigenic cells based on GJIC selectively formed between them. In this study, tumorigenic BALB/c 3T3 and rat liver cells were loaded with Lucifer yellow (LY) and co-cultured with non-tumorigenic cells. By irradiation with blue light, only tumorigenic cells containing LY died but not the surrounding non-tumorigenic cells without LY [149]. This study further showed that when dibutyryl cAMP, retinoic acid, fluocinolone acetonide or dexamethasone were used during cell transformation, there was a reduction of transformed BALB/c 3T3 cell foci. These chemicals also increased and established GJIC between tumour cells and surrounding non-tumour cells, suggesting that the effects of chemicals on reversing the phenotypes of transformed cells rely on the establishment or enhancement of GJIC between tumour and normal cells.

Several cholesterol-lowering statin drugs (lovastatin, simvastatin, etc.) are suggested as anticancer reagents and high levels of mevalonate production are documented in various types of malignancies. Therefore, inhibition of the mevalonate producer, β-Hydroxy β-methylglutaryl-CoA (HMG-CoA) reductase, by statins offers a great potential for cancer treatment [150]. An earlier study shows that lovastatin increases GJIC in transformed E9 mouse lung carcinoma cells through the inhibition of PKC, although Cx43 expression and phosphorylation are not affected [151]. Moreover, apigenin, a flavonoid and lovastatin that is known to increase GJIC enhances bystander effect of the herpes simplex virus thymidine kinase/ganciclovir with reduction of cancer cell recovery on MCA38 adenocarcinoma cells, while neither chemical alone has such effect [152]. In vivo injection of both chemicals achieves 60–70% complete remission of tumour implanted in mice [152]. Simvastatin induced up-regulation of GJIC in Leydig tumour cells and this upregulation sensitized tumour cells to etoposide, a chemotherapeutic drug [153]. Simvastatin inhibited Cx43 phosphorylation by PKC and enhanced Cx43 membrane localization to promote formation of GJs (Ser368 phosphorylation promotes Cx43 internalization). However, a follow up study by the same group reported a protective function of simvastatin against toxicity by cisplatin on normal Sertoli cells [154]. This effect occurs at high cell density where GJIC forms and decreased GJIC by inhibitors or knocking down Cx43 by siRNA attenuates cell protective role of simvastatin. These two studies elucidate differential roles of GJIC by statins in chemotherapy by sensitizing drug effect on cancer cells and ameliorating toxicity in normal cells.

For Cx43 Hcs in cancer development, carbon monoxide (CO), a promising molecule to treat several diseases including cancer has been shown to inhibit their function [155]. CO donors inhibit Hc uptake in tumour cell lines (MCF-7 and HeLa cells) expressing exogenous Cx43 or Cx46 [156]. However, in general, scarce information is currently available describing the involvement of Cx Hcs in cancer cells.

Cxs can directly mediate the effect of chemotherapeutic drugs on cytotoxicity and apoptosis of cancer cells. Upregulation of Cx43 by cisplatin improves its resistance in a mesothelioma cell line (H28) [157]. GJIC inhibition fails to abrogate this effect but it is Cx43-dependent through the suppression of c-Src activation. Cx43 is increased in H28 cells by sunitinib treatment, which promotes apoptosis via the inhibition of receptor tyrosine kinase (RTK) signalling. This effect is likely to be mediated through direct interaction of Cx43 with an apoptotic related protein, Bax [158]. The Cx43 enhanced apoptotic effect of sunitinib was via enhancement of activation of Bax localized at the mitochondrial membrane and the phosphorylation of c-Jun N-terminal kinase (JNK) [159]. Several studies focus on the strategy of enhancing Cx expression in cancer cells. *Ganoderma lucidum*, an herbal mushroom known to inhibit tumour growth can increase Cx43 expression as well as vascular endothelial growth factor (VEGF) and inhibit growth of human ovarian cancer cells [160]. Such effect was abrogated by knocking down Cx43 expression. The bioactive substance sulforaphane inhibits cancer stem cells in aggressive pancreatic ductal adenocarcinoma through increased Cx43 and E-cadherin expression [161]. This treatment also inhibits the cancer stem cell markers c-Met and CD133, alters activation of several kinases and substrates, Glycogen synthase kinase 3 (GSK3), JNK and PKC and enhances GJ channels. Therefore, chemicals that can enhance GJs and Cx expression exhibit a high potency in suppressing cancer cell proliferation and tumour growth.

#### 4.1.2. Connexin-Targeting Strategies in Potential Cancer Therapy

In recent years, several Cx mimetic peptides that reproduced portions of Cx sequences have been widely used in basic research as well as preclinical and therapeutic development [145]. Cx43-GJIC is decreased in breast cancer cells and efforts have been made to restore GJIC in these cells. αCT1, a mimetic peptide that targets CT domain of Cx43 can sustain and enhance GJIC function and has shown a great promise in promoting wound healing in skin by reducing scar formation [162]. A recent study shows that this peptide enhances Cx43 GJIC and reduces proliferation or survival of MCF7 and MDA-MB231 breast cancer cells but has no effect on MCF10A non-transformed cells [163]. A combination of αCT1 with tamoxifen or lapatinib augmented their effects on oestrogen receptor-positive MCF7 or Her2-positive BT474 breast cancer cells. Furthermore, treatment with αCT1 peptide sensitized human O-6-methylguanine-DNA methyltransferase (MGMT)-deficient and chemotherapeutic agent temozolomide (TMZ)-resistant glioblastoma (GBM) cells and combined treatment with the peptide and TMZ further incur autophagy and apoptosis of TMZ-resistance GMB cells [164]. A recent study shows that a cell-penetrating Cx mimetic peptide, TAT-Cx43(266-283) inhibits c-Src and focal adhesion kinase (FAK), upregulates phosphatase and tensin homology and reduces the growth, migration and survival of glioma stem cells (GSCs) from patients [165]. A Cx43 mimetic peptide juxtamembrane 2 (JM2) that is based on the Cx43 microtubule-binding domain inhibits Cx43 trafficking to the cell surface by promoting microtubule polymerization and reduces Hc numbers in the membrane for proinflammatory function. The authors imply that this peptide may have therapeutic value in treating proliferative diseases and cancer [166]. However, it is important to note that the recovery of GJIC does not consistently entail normalization of the tumour cells.

There are several reports concerning use of antibodies against Cxs. When a labelled monoclonal antibody against the second Cx43 extracellular loop domain was intravenously injected into rats with intracranial C6 glioma, antibody signals were detected in reactive, glial fibrillar acidic protein (GFAP)-positive astrocytes [167]. PEGylated immunoliposomes carrying monoclonal antibodies against GFAP and the above-described Cx43 monoclonal antibody were detected at the periphery of the glioma using either fluorescent or a paramagnetic probe [168]. These studies imply that these antibodies could potentially be used for targeted delivery of drugs to the zone of high-grade gliomas. Furthermore, magnetic resonance imaging data show that weekly administration of this Cx43 antibody at a dose of 5 mg/kg significantly reduces low-differentiated glioma volume and increases lifespan with a full recovery without delayed relapses in 19% animals [169]. Both Cx43 and brain-specific anion transporter (BSAT1) are preferably expressed in the brain tumour and peritumoral areas. Cisplatin-loaded nanogel conjugated with monoclonal Cx43 antibody [170] and BSAT1 was used to treat rats bearing tumours and the median survival was greater than control groups [171]. Vector nanogels seemed to reduce systemic toxicity of cisplatin [170]. Intriguingly, a combination of this Cx43 antibody with TMZ completely abolishes the antitumor effect of this antibody while combination treatment with γ-irradiation greatly inhibits tumour development and prolongs survival median to 60 days versus 38 days [172]. Recently, a magnetic resonance imaging (MRI) study further shows that uptake of Gd-based contrast agent with the same monoclonal Cx43 antibody is more than 4 times higher than nonspecific IgG-contrast agent and this Cx43 antibody conjugated agent markedly enhances visualization of glioma in vivo [173]. Although the specific molecular mechanism of this antibody is unknown, this Cx43-targeting monoclonal antibody could be developed as a potential drug and/or diagnostic agent for glioma therapies.

Finally, recombinant lentiviruses carrying siRNA were used to knockdown Cx37 expression in subcutaneous gastric tumours in mice [174]. Reduced levels of Cx37 are associated with higher apoptotic index of tumour cells in vivo. Cx46 is also detected in GBM cancer stem cells, while Cx43 is predominantly expressed in non-stem cells [175]. Besides Cx43, Cx46 is shown to express in GBM cancer stem cells (CSCs) that forms GJIC, while Cx43 is present in non-CSCs. During cancer differentiation, Cx46 is reduced associated with an increase of Cx43 and knocking down Cx46 by short hairpin RNA (shRNA) reduces stem cell maintenance.

Drug resistance is a major challenge for cancer treatment. Cisplatin is a commonly used chemotherapeutic agent for advanced non-small cell lung cancer but prolonged treatment leads to resistance due to development of EMT [119]. Overexpression of Cx43 reverses EMT and cisplatin resistance while Cx43 deletion initiates EMT and drug resistance in human lung cancer cell line A549. Patients with GBM, an aggressive adult primary brain tumour with poor prognosis, develop resistance to TMZ chemotherapy. In contrast to the situation in lung cancer, Cx43 is increased with the formation of GJIC in the resistant tumour cells and this increase is induced by epidermal growth factor receptor (EGFR) activated JNK-ERK1/2-AP-1 signalling [176]. Moreover, Cx43 expression in human glioma cells enhances resistance to TMZ via a mitochondrial apoptosis pathway by the reduction in Bax/Bcl-2 ratio and the release of cytochrome C [177]. Consistently, a recent study [178] showed that TMZ-resistant subline of U251 human GBM cells exhibited elevated Cx43 level compared to parental U251 cells, which was companied with increased EMT markers including vimentin, N-cadherin and β-catenin and decreased cell migration, monocyte adhesion and levels of vascular cell adhesion molecule (VCAM)-1. These studies suggest that depending upon cancer types, Cx43 expression and GJIC could be involved in either promoting or inhibiting sensitization of resistant cells to the chemotherapy. However, the underlying mechanisms remain elusive.

Recently, a new paradigm was proposed based on the data obtained in chronic inflammatory disorders and trauma in the eye that protecting cancer vasculature leads to reduced tumour hypoxia and promote survival of normal cells [179]. Given that Cx43 Hcs are involved in vascular leakage and endothelial cell death [180], modulation of these channels may provide an alternative for cancer treatment. Together, with advanced understanding of the mechanism of Cx channels in various types and stages of cancer development and metastasis, new lines of drugs that target them in cancer therapy are moving closer to reality.

### 4.2. Pannexin Channels in Potential Cancer Therapeutics

A great progress has been made in recent years for our understanding of Panx biology and physiology. However, compared to that of Cxs, the potential therapeutic application of Panxs in cancer is still limited.

#### 4.2.1. Pannexin Channel Activation and Potential Cancer Therapy

Panx channels mediate ATP release and anti-tumour immune responses are associated with such a release from apoptotic cancer cells to engage P2 purinergic receptor signalling in leukocytes. A study shows that apoptotic reagents activate Panx1 channels via caspase-3 cleavage, which leads to ATP release in Jurkat T cell acute lymphocytes in chemotherapeutic drug-induced apoptosis [181,182]. Panx1 level is much higher in leukemic T lymphocytes than untransformed T lymphoblasts. Interestingly, chemotherapeutic drugs also cause ATP release with inhibition of caspase activation, which implies a Panx-independent mechanism. This study suggests that Panx1 channels and ATP release may mediate paracrine interaction between dying tumour cells and leukocytes in anti-tumour responses. A follow up study by the same group shows that activation of Panx1 channels by ATP is determined by expression level of particular ectonnucleotidases in tumour cell variants in Jurkat cell lines with and without the Fas-associated death domain (FADD) or receptor-interacting protein kinase 1 (RIP1) cell death regulatory proteins [183]. They noticed that robust levels of extracellular ATP/AMP were accumulated in apoptosis-deficient cells, not in apoptotic cells with the activation of Panx1 channels in response to chemotherapeutic drugs. Panx1 channel assists in accumulating immune-stimulatory ATP versus immunosuppressive adenosine within the tumour microenvironment. In support of the role of ATP and Panx1 channels in mediating immune response, a very recent study shows that ATP increases migration of dendritic cells through the activation of Panx1 channel and P2X7 receptor (P2X7R) [183]. In this study, they show that ATP actives P2X7R, which leads to opening of Panx1 channels and consequently results in more ATP release, re-organization of the actin cytoskeleton and faster migration of dendritic cells. Additionally, in vivo data show that Panx1 channels are required for the homing of dendritic cells to lymph nodes but not for maturation. Therefore, given that ATP acts as danger signal that recruits phagocytes including dendritic cells to cancer sites, activation of Panx channels through therapeutic drugs could hinder tumour growth and metastasis. Moreover, an US Food and Drug Administration (FDA)-approved anti-parasitic drug, Ivermectin allosterically regulates P2X4 receptors in breast cancer cells through opening of the P2X4/P2X7-gated Panx1 channels, which is associated with ATP release and consequently, cancer cell death [184]. Additionally, Ivermectin induces activation of autophagy and enrichment of inflammation mediators, ATP and high-mobility-group B (HMGB), suggesting that modulation of purinergic receptor signalling could be used as a platform for cancer immunotherapy [185].

#### 4.2.2. Pannexin in Potential Cancer Diagnosis

A clinical report shows high relative expression of Panx3 in a patient with primary cutaneous sweat gland carcinomas with histologic features of a high-grade osteosarcoma [186]. By using quantitative trait loci (QTL) analysis, sequence comparison between strains and gene network analysis, this report links both body mass index (BMI) and tumorigenesis with Panx3 as a candidate gene in a genetically heterogeneous mouse model with carcinogen-induced cancer. A mutation encoding a truncated Panx1 (1–89) was identified which was enriched in highly metastatic breast cancer cells [143]. This truncated form of Panx1 further enhanced ATP release. In contrast to general belief of Panx channels in promoting cancer cell death, this paper suggests that ATP release by Panx1 suppresses deformation-induced apoptosis through P2Y receptor signalling and inhibition of Panx1 channels could reduce the efficiency of breast cancer metastasis. This could be partially explained by excess release of ATP by mutated Panx1 channels. Panx1 is present in skin melanocytes and is upregulated during melanoma tumour progression and tumorigenesis [88]. Knockdown of Panx1 in tumour cells decreases tumour cell growth, which indicates Panx1 as a potential target for treating melanoma. More studies are required to assess the expression levels of Panx subtypes in various types and stages of cancer.

#### 4.2.3. Pannexin Channels in Pain Management Related to Cancer Treatment

Repeated treatment with the chemotherapeutic drug oxaliplatin is limited due to the development of a neuropathic pain in cancer patients. Functional recruitment of Panx1 mediates the increase of P2X7Rs in cerebrocortical nerve terminal in oxaliplatin-treated rats. Moreover, P2X7R antagonists and Panx1 inhibitors, Erioglaucine and ^10^Panx peptide reverts neuropathic pain caused by oxaliplatin, while Panx1 inhibitors do not interfere the cytotoxic effect of oxaliplatin on human colon cancer cells HT-29 [187]. Consistently, a recent study shows that Panx1 expressed in immune cells plays a critical role for pain-like effects after nerve injury and this response is abrogated in Panx1 gene deficient mice [188]. These studies suggest that therapeutic modulation of Panx1 could be useful for treating neuropathic pain associated with cancer and cancer treatment.

## 5. Discussion and Conclusions

The involvement in cancer of GJs and their structural proteins, the Cxs, is a long story [4]. It rose just after the discovery of these particular intercellular junctions, which appeared to be absent in cancer cells. These very first observations suggested that the lack of GJIC could contribute to the lack of cell growth control which characterizes tumorigenesis [18]. Therefore, cell growth control was assumed to be one of the fundamental roles played by GJs. However, if this implication was assumed fifty years ago, the precise molecular mechanisms controlling cell growth came very late and are still unclear. There is a kind of paradox between the amount of observations accumulated for decades confirming a possible role of GJs as guardians of cellular homeostasis and replication and the lack of sufficient evidence explaining such a phenomenon.

Indeed, despite few exceptions, all kinds of observations were suggesting that the lack of GJs or Cxs is correlated to the lack of cell growth control and vice versa This consensus was supported by observations collected from a tremendous variety of models (cancer cell lines, primary cultures of tumour cells, in situ from biopsies, Cx-cDNA transfected cells, Cx-KO mice, chemical treatments decreasing or upregulating GJIC, etc.) whatever the species origins [32]. However, despite this consensus of observations, the assumption that GJs and Cxs were so-called tumour suppressors was not fully supported by several facts. First, GJs and Cxs did not behave as classical tumour suppressors since Cx gene mutations never appeared in tumours as commonly shown as for p53, Rb and so forth [32]. Second, no clear molecular mechanisms underlying the growth control that GJs and Cxs could exert has been established contrary to what was observed with classical tumour suppressors. These two aspects probably restricted GJs and Cxs to be considered as a real hallmark of cancer despite all the consensus studies we mentioned above [189]. The few molecular mechanisms that could explain the cell growth control exerted by GJs and Cxs seems to be “diffuse” and not so straightforward as growth signalling pathways described for oncogenes and tumour suppressors. Indeed, the involvement of Cxs in cell growth control is not clear at the molecular level and appears to be either GJIC-dependent or not. When this involvement was found to depend on GJIC, Cxs permit the intercellular diffusion of metabolites acting on cell growth control (i.e., Cx26 and the diffusion of cAMP, all along the cell cycle phases in HeLa cells) [44]. When this growth control is GJIC-independent, Cxs seem to act through their CT domain as a sequestrator preventing the nuclear translocation of cell growth regulators [53]. By comparing to our knowledge about cell growth control, Cxs seems to be a “helper” instead of a master regulator of cell growth control. Hopefully, future studies will bring more clear-cut information about the real involvement of GJs and Cxs in cancer cell growth [190].

In addition, we have also to consider that exceptions were observed in the consensus supporting the parallel between GJIC and cell growth control. These exceptions, supported by experimental observations, led to the hypothesis that Cxs could be protumoral actors when expressed at late stage of cancer progression. Indeed, from about twenty years ago, it appeared that Cxs could favour migration and invasion of cancer cells and participate to their dissemination [2,94]. A new wave of data then confirmed this new assumption that Cxs are actively involved in the late stages of carcinogenesis and participate to the aggressiveness of solid tumours. Very interestingly, from this more recent domain of investigation, Cxs were shown to play a role not only on migration and invasion of cancer cells but also on metastasis development by acting on intravasation, extravasation and dormancy of the metastatic cells. Within a few years, the Cx cancer statute has been changed then from tumour suppressor to tumour enhancer. Contrary to what appears at a first glance, this is not contradictory since an inverted correlation is often observed between cell proliferation and invasion capacity [191].

As for cell growth control, the molecular mechanisms underlying the involvement of Cxs in cell migration are not very clear. Once again, Cxs seem to control cell migration either through channel-dependent or –independent mechanisms. In the first case, the establishment of heterologous GJIC between cancer cells and cells of the tumour microenvironment may increase motility (such as glioma cells communicating with astrocytes) and further, helps to intravasation and extravasation [124,125,126,127,192]. When isolated in extracellular matrix, Cxs act on motility through GJIC-independent mechanisms by its CT domain. This has been particularly studied for Cx43 and even if the precise molecular events are not elucidated yet, it seems that the CT domain is involved by interacting with the actin cytoskeleton and helps to manage directional migration of the cancer cells [193]. Interestingly, such a phenomenon would not be pathological by itself since this process is present in migrations occurring in normal situations such as embryogenesis (neuron precursors migrating to the cortex) and leucocyte migration [194,195,196]. Other data also suggest that Cxs could be involved in formation of invadopodia and secretion of proteases during invasion process and also in metastasis targeting [132]. The molecular processes of all these phenomena are far from being to be elucidated. More data are needed to explain at the molecular level how Cxs can control cancer cell invasion and metastasis. These data are necessary for targeting Cxs to prevent eventually cancer invasion. This is of fundamental importance when considering that the majority of cancer deaths are the consequence of metastasis [1].

To conclude, there are globally sufficient data showing that Cxs are involved in carcinogenesis, especially in the progression of solid tumours. However, despite these data, the molecular mechanisms of the Cx involvement in carcinogenesis are not sufficiently elucidated yet. This lack of knowledge limits to use them as general therapeutical targets for cancer control. Moreover, the multifunctional sides of Cxs able to act as mediator of GJIC, through their interactome or even as Hcs make difficult to define their real implication in cancer. In addition, the similarity of Panxs with Cx Hcs adds another complexity to this area of research since this family of proteins seems to share functions with Cxs both in cell proliferation control and invasion. Facing this complexity, the only way to decipher the real impact of Cxs or Panxs in the cancer cell behaviour is to consider their involvement specifically in particular types of tumours but not globally [190]. One strategy could be by increasing in situ observations in order to localize precisely Cx/Panx expressions in the complex heterogeneity of specific human tumours and reveal the possible links of Cx/Panx localizations with the tumour behaviour. In particular, it could prove definitively the apparently opposed roles of Cxs in cell growth control and in cancer cell invasion through their differential expression either in the core of the tumour or in its invasive edges [111]. Therefore, due to uniqueness of the action of subtypes of Cxs and Cx channels on various types and stages of cancers, therapeutic approaches ought to be developed based on precise mechanism elucidated with more targeting approaches. This aligns with the current trend of drug development in treating cancer with precision medicine.

## Figures and Tables

**Table 1:** Selected representative examples of changes in connexins during tumour progression and metastasis.

TISSUE	ORGANISM	CONNEXIN	REGULATION	REFERENCE
***PRECANCERS AND PRIMARY TUMOURS***				
**PANCREATIC DUCTAL ADENOCARCINOMA**	Mouse	Cx43	Increased levels Changes in phosphorylation	[89]
**CERVICAL CANCER**	Human	Cx26, Cx30, Cx43	Loss of connexin expression	[95,96,97]
**BREAST CANCER**	Human	Cx26, Cx43	Loss of Cx43 gap junctions	[98,99,100,101]
**PROSTATE CANCER**	Human	Cx32, Cx43	Decreased expression	[102]
**COLON CANCER**	Human	Cx32, Cx43	Gradual loss of expression	[92]
**MELANOMA**	Human	Cx26, Cx30	Increased expression	[103]
***PRIMARY TUMOUR TO METASTASIS***				
**BREAST CANCER**	Human	Cx26, Cx43		[101,104,105,106,107,108]
**BRAIN**	Human, rat Human	Cx30 Cx43	Reduced expression	[109] [110,111]
**PROSTATE**	Human cell lines	Cx43	Increased Cx43 associated with increased invasion	[112]
**LIVER**	Rat cell lines Human	Cx43 Cx26	Cx43 overexpression High expression	[113] [114]
**MELANOMA**	Human Human cell lines	Cx26	Increased expression	[103,115] [116]
